# Lung Function Impairment, Associating Hyperinflation with Impaired Diffusion Capacity and Transfer Coefficient, Is a Risk Factor for Hip Osteoporosis in Patients with Chronic Obstructive Pulmonary Disease

**DOI:** 10.3390/jcm12062383

**Published:** 2023-03-20

**Authors:** Olivier Malaise, Cécile André, Caroline Van Durme, Laurence Seidel, Florence Schleich, Renaud Louis, Michel Malaise, Clio Ribbens

**Affiliations:** 1 Department of Rheumatology, University Hospital of Liège, 4000 Liège, Belgium; 2 Department of Physical Medicine and Rheumatology, Centre Hospitalier Chrétien, 4000 Liège, Belgium; 3 Department of Internal Medicine, Maastricht University Medical Centre, 6229 Maastricht, The Netherlands; 4 Biostatistics and Research Method Center, ULiège and University Hospital of Liège, 4000 Liège, Belgium; 5 Department of Respiratory Medicine, University Hospital of Liège, 4000 Liège, Belgium

**Keywords:** osteoporosis, chronic obstructive pulmonary disease, emphysema, screening

## Abstract

Chronic obstructive pulmonary disease (COPD) is a risk factor for osteoporosis. Our objective is to determine if functional indices associated with emphysema on pulmonary function tests (DLCO—diffusion capacity of the lung for CO-; DLCO/AV—DLCO corrected for alveolar volume- and TLC—total lung capacity), considered alone or together, can identify COPD patients with osteoporosis. Methods: 90 COPD patients underwent dual-energy X-ray absorptiometry (DEXA) and pulmonary function tests. Results: 26% of the COPD patients were osteoporotic. In univariate analysis, each functional parameter associated with emphysema, analyzed separately, was not associated with osteoporosis. In contrast, patients with hyperinflation associated with impaired diffusion capacity and transfer coefficient, defined by the association of the three functional indices (DLCO < 70%, DLCO/AV < 80% and CPT > 115%), had significantly more osteoporosis at the total hip (OR: 5.9, CI: 1.5–23.8, *p* = 0.013). In multivariate analysis, this phenotype was confirmed as an independent factor associated with hip osteoporosis. In contrast, COPD airway obstruction severity, based on FEV_1_ (%), was not associated with osteoporosis. A lower BMI, female gender and age were also identified as osteoporosis risk factors. Conclusions: COPD patients with hyperinflation associated with impaired diffusion capacity and transfer coefficient are at higher risk for osteoporosis. Pulmonary function tests associated with emphysema detection can help to identify COPD patients with osteoporosis, in addition to the classical risk factors.

## 1. Introduction

Chronic obstructive pulmonary disease (COPD) is a heterogeneous lung condition characterized by chronic respiratory symptoms (dyspnea, cough, sputum production, exacerbations) due to abnormalities of the airway (bronchitis, bronchiolitis) and/or alveoli (emphysema) that cause persistent, often progressive, airflow obstruction [1]. COPD is also characterized by systemic inflammation and extra-pulmonary manifestations, such as osteoporosis [2]. As in the general population, osteoporosis is linked with mortality and morbidity in COPD patients: lower T-scores are associated with a 5% increase in mortality [3], fractures are linked with higher mortality [4] and retrospective analysis of our local COPD cohort identifies osteoporosis as a mortality risk factor among patients receiving long-term oxygen therapy [5]. Morbidity is increased with higher hospitalization rates [4] and a decline in lung function is associated with vertebral fractures [6,7]. Despite these data, osteoporosis detection is still an unmet problem and studies confirm that the rate of COPD patients treated for osteoporosis is low with regard to osteoporosis prevalence [8,9].

It is well established that osteoporosis prevalence is higher in COPD patients than in the healthy population [10], and numerous risks factors of osteoporosis are found in patients with COPD: aging, smoking, physical inactivity, systemic inflammation, malnutrition, low body-mass index (BMI), corticosteroids … [10,11]. COPD patients are heterogenous and have different phenotypes (emphysema, chronic bronchitis, frequent exacerbator …), with variations in terms of demographics, disease characteristics and health outcomes [12]. The emphysema phenotype is described to be associated with more dyspnea [13], more exacerbation [14] and more disease progression [2]. Several studies indicate that CT quantification of emphysema [15,16,17,18] is associated with low bone mineral density (BMD) or osteoporosis, but there is no reliable information about the functional characteristics associated with emphysema based on functional respiratory tests.

Our objective is to analyze whether a higher probability of osteoporosis is found in COPD patients who exhibit functional respiratory tests parameters that reflect emphysema (DLCO -diffusion capacity of the lung for CO- <70%; DLCO/AV -DLCO corrected for alveolar volume- <70%; and TLC -total lung capacity- >115%). We also evaluated the hypothesis that a combination of these three functional parameters could help to identify COPD patients with osteoporosis.

## 2. Materials and Methods

### 2.1. Subjects

Ninety consecutive ambulatory COPD patients were evaluated by dual-energy X-ray absorptiometry (DEXA) and pulmonary function tests. The diagnosis of COPD had to be established by a respiratory physician and patients had to be followed in pneumology consultation.

### 2.2. Measurements

All subjects filed a questionnaire about their smoking habits, alcohol consumption, fracture history and use of corticosteroids (current or previous use, systemic and/or inhaled). All bone examinations were performed by the same Discovery A DEXA system (Hologic^®^, Bedford, MA, USA), with lumbar spine (L1–L4) and left total hip and femoral neck analysis. T-score values were considered osteoporotic if ≤−2.5. Standardization procedures were performed according to the International Society for Clinical Densitometry. In particular, quality controls with phantom were performed daily to ensure that these values were located at a maximum of ±1.5% of the mean value of calibration. The total bone mineral density (BMD) coefficient of variation (CV) of the DEXA (Hologic^®^, Bedford, MA, USA) = 1.0%.

The same day, patients underwent functional respiratory tests with spirometric measurements, lung volume and lung diffusion analysis: FEV_1_ (fraction of expired volume in 1 s) (%) stratification for COPD severity (mild if >80%; moderate if ≥50 and ≤80; severe if ≥30 and <50; and very severe if <30); DLCO (diffusion capacity of the lung for CO); DLCO/AV (DLCO corrected for alveolar volume); and TLC (total lung capacity) for parameters associated with functional emphysematous. DLCO, DLCO/AV and TLC were considered pathological if <70%, <80% or >115%, respectively.

Approval was submitted to the local ethical committee of the University Hospital of Liège but was not mandatory because all examinations performed (functional respiratory test and DEXA) were part of the standard of care of COPD management. All procedures performed in studies involving human participants were undertaken in accordance with the ethical standards of the institutional research committee and with the 1964 Helsinki declaration and its later amendments or comparable ethical standards.

### 2.3. Statistical Analysis

Results are presented as mean ± standard deviation (SD) or as median (minimum–maximum) for continuous variables and as frequency tables for qualitative variables. Univariate logistic regression models investigated the relationship between osteoporosis risk and demographic/respiratory variables (odds ratio ± 95% confidence interval). Multivariate logistic regression model (including the following variables: gender, age, BMI, corticosteroids (chronic use), corticosteroids history of use, active smoking, FEV_1_ and association of DLCO < 70%, DLCO/AV < 80% and TLC > 115%) was applied on these factors Results were considered significant at the uncertainty level of 5% (*p* < 0.05). Calculations were performed using SAS software version 9.4 (SAS Institute, Cary, NC, USA).

## 3. Results

### 3.1. Demographics

Ninety COPD patients were included (Table 1). Of these, 62.2% were male, with a mean (±SD) age of 63.4 (±10.1) years and a mean BMI of 24.1 (±5.2) kg/m^2^. Forty (44%) were active smokers. In total, 68.9% had a chronic use of corticosteroids: 62.2% had only chronic inhaled treatment and 6.7% also received more than 2 systemic courses per year. Finally, 71.1% had a history of glucocorticoid use as defined by the FRAX (current or previous treatment for >3 months at a prednisone dose ≥5 mg/day).

### 3.2. DEXA Results and Pulmonary Function Tests

Osteoporosis in at least one of the three sites was identified in 23 patients (25.6%). At each anatomical location, osteoporosis rates were as follows: 15.6% at lumbar spine, 15.7% at femoral neck and 13.5% at total hip. BMD and T-scores are shown in Table 2. COPD patients were characterized by pulmonary function tests (Supplementary Data: Table S1). For lung functional parameters associated with emphysema (DLCO, DLCO/AV and TLC), each parameter was first considered separately. We also decided to consider the association of these three functional parameters and created a sub-group of patients (20/80, 25%) exhibiting “DLCO < 70%, DLCO/AV < 80% and TLC > 115%”. Of these patients, seven (35.0%) had global osteoporosis. At the subtype level, four (20.0%) had L1L4 osteoporosis, four (20.0%) had femoral neck osteoporosis and six (30.0%) had total hip osteoporosis.

### 3.3. Osteoporosis Risk Factors: Univariate Analyses

In univariate analysis, the severity of airway obstruction (FEV_1_) was not associated with osteoporosis diagnosis (Table 3). While each functional parameter associated with emphysema (DLCO, DLCO/AV, TLC), taken separately, was not associated with a diagnosis of osteoporosis, patients that had associated hyperinflation with impaired diffusion capacity and transfer coefficient (DLCO < 70%, DLCO/AV < 80% and TLC > 115%) were more likely to exhibit total hip osteoporosis, with an OR of 5.89 (IC95%: 1.46–23.77, *p* = 0.013). No significant association was observed for the lumbar spine or the femoral neck. A higher BMI was less likely to be associated with osteoporosis at all the sites analyzed (OR:0.74, 0.61–0.89, *p* = 0.002; OR:0.82, 0.70–0.96, *p* = 0.016; OR:0.66, 0.51–0.84, *p* = 0.0007 for lumbar spine, femoral neck and total hip, respectively), while active smoking was more likely to be associated with osteoporosis (OR:5.94, 1.52–23.11, *p* = 0.01; OR:3.97, 1.14–13.83, *p* = 0.031 for lumbar spine and femoral neck, respectively). Association with glucocorticoid use was restricted to total hip osteoporosis.

### 3.4. Osteoporosis Risk Factors: Multivariate Analyses

The multivariate analysis for osteoporosis risk included eight variables: gender, age, BMI, chronic use of corticosteroids, history of corticosteroids use, active smoking, FEV_1_ and association of “DLCO < 70%, DLCO/AV < 80% and TLC > 115%” (Table 4). COPD severity (FEV_1_) was not associated with osteoporosis. Hyperinflation with impaired diffusion capacity and transfer coefficient remained a significant risk factor for total hip osteoporosis: patients that associated DLCO < 70%, DLCO/AV < 80% and TLC > 115% were more likely to exhibit total hip osteoporosis (OR, IC95%: 12.1, 1.1–138.8, *p* = 0.045). A higher BMI was more likely to be associated with less lumbar spine and total hip osteoporosis, while male gender was also less likely to be associated with osteoporosis at the femoral neck and total hip. A higher age was linked with total hip osteoporosis.

## 4. Discussion

In this study, we analyzed the association between functional lung parameters and bone mineral density in COPD patients. When we considered separately the functional parameters associated with emphysema (DLCO, DLCO/AV or TLC), none of these three factors was associated with osteoporosis. However, hyperinflation with impaired diffusion capacity and transfer coefficient (the association of DLCO < 70%, DLCO/VA < 80% and TLC > 115%) was as an independent risk factor for hip osteoporosis, even after adjustment for clinical and spirometric factors.

The influence of pulmonary emphysema on bone metabolism had already been analyzed in previous studies. In four different studies [15,16,17,18], authors found a link between pulmonary emphysema and low BMD, but pulmonary emphysema was assessed by chest CT, which is less accessible than functional respiratory tests and not routinely repeated in daily clinics. Ohara et al. have confirmed that this association was independent of BMI and FEV_1_ [18]. All these studies were based on CT data and supported the “holes in the lung, holes in the bone” theory. None of these studies analyzed the emphysematous parameters using functional respiratory tests. There are a few exceptions, with Fouda et al. in 2017, who also made functional respiratory tests to determine emphysematous patients among 52 COPD patients [19]. Twenty-seven were emphysematous (definition based on DLCO/AV < 80%), but there was no difference in terms of BMD. Another study found no influence of DLCO/AV or TLC and T-score, but included only 50 patients and none with a mild disease stage [20]. In our study, emphysema was investigated with respiratory functional tests, available in daily practice. Complete functional emphysema parameters using pulmonary respiratory tests (hyperinflation with impaired diffusion capacity and transfer coefficient) were associated with osteoporosis at the total hip in univariate analysis and confirmed in multivariate analysis. We have also analyzed two of three parameters, but no correlation was found. Fouda et al. considered that emphysema was not a determinant of osteoporosis and suggested a link between FEV_1_ and emphysematous status to explain that emphysema was significant in some studies [19]. In our study, COPD severity of obstruction (analyzed with FEV_1_%) was not a risk factor for bone disease associated with COPD and could not interfere. COPD severity has been associated with bone disease in individual studies (e.g., lower T-scores in patients with GOLD D COPD, links between BMD and FEV_1_ (%) [20]), but not in meta-analyses. In our population, FEV_1_ was not associated with osteoporosis diagnosis or demineralization, meaning that COPD obstruction severity is not a significant factor to consider in osteoporosis risk.

In our cohort, lumbar spine BMD density was not associated with the spirometric diffusion value, while there was a correlation for the total hip. A first explanation can be that tobacco, which is the main driver of COPD, could have a direct negative effect on cortical bone, rather than on trabecular bone (negative correlation between femoral neck BMD and both cigarette consumption (per-day) and packets-years according to [21]; association between smoking and reduced cortical thickness in young men according to [22]). On the other hand, lumbar spine analysis is less reliable in the elderly, with overestimation in the case of vertebral fracture or spine osteoarthritis (the mean age of our cohort was 64.5 years, with 80 for the oldest patient).

Our patients with higher BMI had less osteoporosis. This is consistent with the pooled data by Chen et al. that associated BMI < 18.5 kg/m^2^ with an OR of having osteoporosis of 4.26 and an increase of BMI by 1 kg/m^2^ with an OR of 0.8 [9]. We previously identified low BMI as one of the most potent drivers of osteoporosis in the overall population of our hospital [23]. This is of particular importance in the COPD population with regard to the high rate of low BMI and sarcopenia (sarcopenia being more important in COPD patients with osteoporosis or osteopenia than in COPD patients with normal BMD [7]).

Osteoporosis prevalence was 25.6% in our COPD population. Two recent meta-analyses [10,11] estimated this prevalence at 38 and 37%, more than our data. This could be related to geographic particularities since a COPD cohort coming from a neighboring country showed a very close incidence to ours (21%) [8]. Lower osteoporosis prevalence could perhaps be explained by the recruitment of our population, which was ambulatory with regular follow-up. The ambulatory COPD population could be in better health than people with sub-optimal follow-up.

In conclusion, our data show that lung function impairment, associating hyperinflation with impaired diffusion capacity and transfer coefficient (DLCO < 70%, DLCO/AV < 80% and TLC > 115%), is an independent risk factor for hip osteoporosis. In contrast, the severity of airway obstruction was not associated with bone density parameters. Pulmonary function tests with emphysema detection could help to identify patients that need osteoporosis screening.

## Figures and Tables

**Table 1:** Demographic and clinical data for the 90 COPD patients.

Variable	N	Mean	SD	Min	Median	Max
Age (year)	90	63.4	10.1	40.0	64.00	83.0
Height (cm)	90	167.4	9.1	149.0	167.00	185.5
Weight (kg)	90	68.1	17.3	35.0	66.00	118.5
BMI (kg/m^2^)	90	24.1	5.2	14.5	23.40	44.0
Variable	N	Categories			N (%)	
Sex	90	Male			56 (62.2)	
Smoking	90	No (non-smoker)			1 (1.1)	
		No (ex-smoker)			49 (54.4)	
		Yes (active smoker)			40 (44.4)	
Alcohol	90	No			28 (31.1)	
		Yes			30 (33.3)	
		Occasional			32 (35.6)	
Corticosteroids (history of use)	90	No			64 (71.1)	
		Yes			26 (28.9)	
Corticosteroids (chronic use)	90	No			28 (31.1)	
		Yes (total)			62 (68.9)	
		Yes (inhaled only)			56 (62.2)	
		Yes (also systemic)			6 (6.7)	
Fracture history	90	No			41 (45.6)	
		Yes			49 (54.4)	
Hip fracture history	90	No			85 (94.4)	
		Yes			5 (5.6)	
Vertebral fracture history	90	No			83 (92.2)	
		Yes			7 (7.8)	

“Corticosteroids history of use” was defined as described in the FRAX algorithm (current or previous treatment for >3 months at a prednisone dose 35 mg/day). N = number. SD = standard deviation.

**Table 2:** Description of bone mineral density, T-scores and DEXA diagnosis (normal, osteopenia or osteoporosis). N = number. SD = standard deviation. BMD = Bone Mineral Density.

Variable	N	Mean	SD	Min	Median	Max
BMD lumbar spine (g/cm^2^)	90	0.937	0.181	0.6	0.92	1.5
BMD femoral neck (g/cm^2^)	89	0.687	0.123	0.4	0.68	1.0
BMD total hip (g/cm^2^)	89	0.819	0.151	0.4	0.82	1.1
T-score lumbar spine	90	−0.959	1.647	−4.2	−1.30	3.9
T-score femoral neck	89	−1.547	0.996	−4.3	−1.60	0.7
T-score total hip	89	−1.258	1.025	−4.2	−1.30	0.8
Variable	N	Categories		N (%)		
Global diagnosis	90	Normal		18 (20.0)		
		Osteopenia		49 (54.4)		
		Osteoporosis		23 (25.6)		
Lumbar spine diagnostic	90	Normal		39 (43.3)		
		Osteopenia		37 (41.1)		
		Osteoporosis		14 (15.6)		
Femoral neck diagnostic	89	Normal		22 (24.7)		
		Osteopenia		53 (59.6)		
		Osteoporosis		14 (15.7)		
Total hip diagnostic	89	Normal		32 (36.0)		
		Osteopenia		45 (50.6)		
		Osteoporosis		12 (13.5)		

**Table 3:** Correlation between clinical/respiratory parameters and osteoporosis diagnosis (univariate analyses).

	Lumbar Spine				Femoral Neck				Total Hip			
	Osteoporosis L1–L4 (vs. Osteopenic/Normal)				Osteoporosis Femoral Neck (vs. Osteopenic/Normal)				Osteoporosis Total Hip (vs. Osteopenic/Normal)			
	OR	CI 95%		*p*-Value	OR	CI 95%		*p*-Value	OR	CI 95%		*p*-Value
Gender (male)	0.39	0.122	1.244	0.11	0.069	0.014	0.335	0.0009	0.16	0.04	0.644	0.0099
Age (year)	0.991	0.936	1.048	0.75	0.997	0.942	1.055	0.91	1.041	0.975	1.111	0.23
BMI (kg/m^2^)	0.735	0.606	0.893	0.002	0.820	0.698	0.963	0.016	0.655	0.513	0.837	0.0007
Corticosteroids (chronic use)	1.154	0.328	4.053	0.82	1.106	0.314	3.895	0.88	2.404	0.49	11.803	0.28
Corticosteroids (history of use)	1.455	0.437	4.847	0.54	2.947	0.915	9.494	0.070	4.274	1.213	15.054	0.024
Active smoking	5.943	1.528	23.105	0.010	3.965	1.137	13.829	0.031	2.968	0.822	10.714	0.097
FEV_1_ (%)	1.014	0.98	1.048	0.43	1.008	0.975	1.042	0.65	0.992	0.957	1.028	0.66
TLC (%)	1.011	0.977	1.047	0.53	1.003	0.969	1.038	0.88	1.029	0.991	1.07	0.14
TLC > 115%	1.154	0.331	4.017	0.82	0.741	0.203	2.706	0.65	2.640	0.680	10.256	0.16
DLCO (%)	1.017	0.976	1.06	0.43	0.99	0.95	1.033	0.65	0.968	0.922	1.015	0.18
DLCO < 70%	2.185	0.496	9.628	0.30	0.463	0.054	3.96	0.48	0.586	0.067	5.101	0.63
DLCO/AV (%)	0.997	0.973	1.021	0.78	0.992	0.967	1.017	0.51	0.972	0.942	1.003	0.081
DLCO/AV < 80%	1.429	0.417	4.889	0.57	0.936	0.269	3.251	0.92	0.525	0.125	2.202	0.38
DLCO < 70%, DLCO/AV < 80% and TLC > 115%	1.625	0.432	6.111	0.47	1.594	0.424	5.997	0.49	5.894	1.461	23.769	0.013

OR = odds ratio. CI = confidence interval. BMI = body mass index. FEV_1_ = fraction of expired volume in 1 s. FVC = forced vital capacity. TLC = total lung capacity. DLCO = diffusion capacity of the lung for CO. AV = alveolar volume. In grey and bold, significant correlation (*p* < 0.05). Lumbar spine: N = 90 (N = 80 for TPC, DLCO, DLCO/AV and association). Femoral neck and total hip: N = 89 (N = 79 for TPC, DLCO, DLCO/AV and association).

**Table 4:** Correlation between clinical/respiratory parameters and osteoporosis diagnosis (multivariate analyses).

	Lumbar Spine				Femoral Neck				Total Hip			
	Osteoporosis L1–L4 (vs. Osteopenic/Normal) (AUC = 0.817)				Osteoporosis Femoral Neck (vs. Osteopenic/Normal) (AUC = 0.872)				Osteoporosis Total Hip (vs. Osteopenic/Normal) (AUC = 0.946)			
	OR	CI95%		*p*-Value	OR	CI95%		*p*-Value	OR	CI95%		*p*-Value
Gender (male)	0.503	0.099	2.549	0.41	0.049	0.006	0.393	0.005	0.034	0.001	0.905	0.043
Age (year)	1.029	0.958	1.105	0.43	1.027	0.950	1.111	0.50	1.110	1.001	1.231	0.047
BMI (kg/m^2^)	0.795	0.635	0.996	0.047	0.850	0.690	1.048	0.13	0.635	0.448	0.899	0.011
Corticosteroids (chronic use)	1.029	0.205	5.156	0.97	0.761	0.129	4.487	0.76	0.866	0.066	11.393	0.91
Corticosteroids (history of use)	1.079	0.181	6.425	0.93	1.366	0.246	7.574	0.72	4.675	0.458	47.727	0.19
Active smoking	4.715	0.990	22.453	0.052	4.856	0.856	27.566	0.074	7.255	0.363	144.891	0.19
FEV_1_ (%)	1.015	0.971	1.061	0.50	0.991	0.942	1.041	0.71	0.968	0.895	1.047	0.42
DLCO < 70%, DLCO/AV < 80% and TLC > 115%	1.194	0.235	6.057	0.83	1.539	0.291	8.150	0.61	12.143	1.062	138.806	0.045

OR = odds ratio. CI = confidence interval. BMI = body mass index. FEV_1_ = fraction of expired volume in 1 s. Lumbar spine: N = 80. Femoral neck and total hip: N = 79.

## Data Availability

The data presented in this study are available on reasonable request from the corresponding author.

## Supplementary Materials

The following supporting information can be downloaded at: https://www.mdpi.com/article/10.3390/jcm12062383/s1, Table S1: Description of pulmonary parameters on functional respiratory tests. N = number. SD = standard deviation. FEV_1_ = fraction of expired volume in 1 s. FVC = forced vital capacity. TLC = total lung capacity. DLCO = diffusion capacity of the lung for CO. AV = alveolar volume. N = number. SD = standard deviation.
