# Supplementary material for: Lung Function Impairment, Associating Hyperinflation with Impaired Diffusion Capacity and Transfer Coefficient, Is a Risk Factor for Hip Osteoporosis in Patients with Chronic Obstructive Pulmonary Disease
Source: J Clin Med. 2023 Mar 20;12(6):2383. doi: 10.3390/jcm12062383 (PMC10059846; doi:10.3390/jcm12062383)
Supplement: Supplementary file 1 [file jcm-12-02383-s001.zip › jcm-2224754-supplementary.pdf]

Supplementary Materials:

Table S1. Description of pulmonary parameters on functional respiratory tests.

**Variable**	**N**	**Mean**	**SD**	**Min**	**Median**	**Max**
FEV1 (mL)	90	1530.9	562.90	510.0	1430.0	3050.0
FEV1 (%)	90	55.311	17.160	23.0	53.50	93.0
FEV1/FVC (%)	90	56.037	11.322	32.1	55.40	89.7
TLC (mL)	80	6583.3	1468.8	3570.0	6710.0	9860.0
TLC (%)	80	109.99	17.637	68.0	109.50	153.0
DLCO (mmol/Kpa/min)	80	4.256	1.428	1.5	4.03	8.0
DLCO (%)	80	50.963	15.245	15.0	50.50	83.0
DLCO/AV (mmol/Kpa/min/L)	80	1.064	0.361	0.3	1.03	2.0
DLCO/AV (%)	80	76.163	25.705	21.0	75.00	132.0

Variable	N	Categories	N (%)
GOLD Stage	90	1 (FEV_1 > 80%)	8 (8.9)
		2 (FEV_1 ≥50 & ≤80%)	46 (51.1)
		3 (FEV_1 ≥30 & <50%)	31 (34.4)
		4 (FEV_1 <30%)	5 (5.6)
FEV1 (%)	90	<30	5 (5.6)
		≥30 & <50	31 (34.4)
		≥50 & ≤80	46 (51.1)
		> 80	8 (8.9)
DLCO (%)	80	<70	68 (43.3)
		≥70	12 (41.1)
DLCO/AV (%)	80	<80	22 (24.7)
		≥80	53 (59.6)
TLC (%)	80	≤ 115	49 (61.3)
		>115	31 (38.8)
DLCO<70%, DLCO/AV<80% & TLC>115%	80	No	60 (75)
		Yes	20 (25)
